# Immunosenescence and lymphomagenesis

**DOI:** 10.1186/s12979-018-0130-y

**Published:** 2018-09-21

**Authors:** Salvatrice Mancuso, Melania Carlisi, Marco Santoro, Mariasanta Napolitano, Simona Raso, Sergio Siragusa

**Affiliations:** 1 Haematology, Biomedical Department of Internal Medicine and Medical Specialties, University of Palermo, Palermo, Italy; 2 Department of Surgical, Oncological and Stomatological Disciplines, University of Palermo, Palermo, Italy

**Keywords:** Lymphoma, Lymphomagenesis, Immunosenescence, Ageing, Cancer

## Abstract

One of the most important determinants of aging-related changes is a complex biological process emerged recently and called “immunosenescence”. Immunosenescence refers to the inability of an aging immune system to produce an appropriate and effective response to challenge. This immune dysregulation may manifest as increased susceptibility to infection, cancer, autoimmune disease, and vaccine failure. At present, the relationship between immunosenescence and lymphoma in elderly patients is not defined in a satisfactory way.

This review presents a brief overview of the interplay between aging, cancer and lymphoma, and the key topic of immunosenescence is addressed in the context of two main lymphoma groups, namely Non Hodgkin Lymphoma (NHL) and Hodgkin Lymphoma (HL). Epstein Barr Virus (EBV) plays a central role in the onset of neoplastic lymphoproliferation associated with immunological changes in aging, although the pathophysiology varies vastly among different disease entities. The interaction between immune dysfunction, immunosenescence and Epstein Barr Virus (EBV) infection appears to differ between NHL and HL, as well as between NHL subtypes.

## Background

Immunosenescence is a peculiar remodeling of the immune system, caused by aging, associated with a wide variety of alterations of immune functions. Mounting biological evidence supports the potential clinical relevance and impact of immunosenescence [1, 2]. Indeed, it is has been implicated in pathophysiology of dementia, frailty, cardiovascular diseases, and it is cause of increased susceptibility to infectious disease, autoimmunity and cancer. Hematological malignancies and lymphoma are diseases that typically affect the elderly, with a median age for the most common lymphoma type, Diffuse Large B-cell Lymphoma (DLBCL), of > 70 years at diagnosis [3]. With the profound changes in demographic profiles of western countries and a steadily rising life expectancy, the number of elder patients with lymphoma is increasing [4, 5].

Although the risk of developing these neoplasms is higher in individuals with inherited predisposition or subjected to environmental risk factors, most cases cannot be associated with identifiable underlying conditions. In fact, lymphomagenesis is proven to be a molecularly complex process resulting in a broad category of different lymphoproliferative disorders. On the basis of histologic features, the pathogenetic events involved in disease initiation and/or progression may vary significantly [6].

Information regarding global burden of immunosenescence in lymphomagenesis is limited and requires a detailed understanding by the type of lymphoma considered, because the mechanism might vary in different specific cases [7].

This review aims to summarize the current state of understanding about the role of immunosenescence and development of lymphoma in older age.

We conducted a systematic research on PubMed, without filtering the results by date, language, or article type. Several studies have shown the potential to address the problem; however, no reports were found of studies in which clinically relevant relationships between the development of lymphoma and the complex underlying immunosenescence were elucidated.

## Aging, cancer and immunosenescence

### Age-related changes and cancer

It is commonly accepted that aging is the main risk factor for major chronic diseases such as cardiovascular diseases, cancer and neurodegenerative diseases [8]. Recently, nine candidate hallmarks of aging were described in relation to genetic, epigenetic and environmental events [Table 1] [9, 10]. The resulting aging process and the aging phenotype, characterized by loss of fitness, create a favorable condition for aberrant neoplastic proliferation [11]. The strong association between cancer and age is supported by epidemiological data, indicating an increased incidence of malignancies in elderly patients. Indeed, about 55% of tumors affect subjects who are over 65 years of age https://seer.cancer.gov/archive/csr/1975_2005/. Aging reflects the sum of all changes accumulating with time by effect of genetic and environmental causes [12, 13]. The more prolonged exposure to carcinogens in older people and the increase in mutational load, epigenetic gene silencing, telomere dysfunction, unrestricted replicative potential, altered stromal milieu, apoptosis evasion, all contribute to an altered environment that promotes neoplastic proliferation [14–17].

**Table 1:** The hallmarks of aging

Genomic instability	
Telomer attrition	
Epigenetic alterations	
Loss of proteostasis	
Deregulated nutrient-sensing	
Mitochondrial dysfunction	
Cellular senescence	
Stem cell exhaustion	
Alterated intercellular communication	

### Biological basis of immunosenescence

Among the spectrum of hallmarks, the changes of the immune response during aging now emerge as an expanding field of research, supported by copious experimental data [18]. Immunosenescence is a complex biological process that occurs in both the innate and adaptive components of the immune system [19]. Most of the effector functions of neutrophils, monocyte/macrophage lineage and natural killer (NK) cells decrease, concomitantly with a basal activation state [20–22]. Chronic antigenic stimulation probably underlies the marked changes in the adaptive immune system [23]. The ultimate consequence is a shift from loss of diversity of the T-cell receptor (TCR) repertoire to an increase in number of exhausted CD28^−^ T cells, and profound functional changes in CD4 T cell subpopulations [8, 24, 25]. Together, these alterations of the innate and adaptive immunity favor the gradual development of a state of chronic inflammatory process called “inflammaging” [26, 27]. It is not perfectly clear if the state of slightly raised inflammatory mediators is really part of immunosenescence or an independent phenomenon with additive effects on morbidity and mortality [28, 29]. To this regard, a basic characteristic of the immune system is plasticity, the capability of immune cells to undergo modification and adapt to different situations [30]. There is limited knowledge on whether immunosenescence is really associated with detrimental clinical outcomes or whether changes in immune parameters of older people reflect adaptive responses to the clinical and immunological history of the subject [31–33]. Therefore, it is necessary to reconsider some conclusions on the basis of many disparate findings in the literature. Against certain generalizations related to immunosenescence, context-dependent immune ageing processes need to be identified, with a detailed understanding of those diseases that are of major health interest [34].

### Immunosenescence and defects in cancer protection

It is well known that both the innate and the adaptive immune system protect the host against carcinogenesis by a process called “immunosurveillance”. By means of this process, the immune cells identify and eliminate cancerous cells before tumor develops [35]. In some cases, the functional ability of immunosurveillance cannot prevent the tumors from escaping and growing because the pressure exerted by the immune system can select many variants of resistant tumor cells. Immunosenescence can be considered as an additional factor which further promotes the tumor’s escape mechanism [36].

Dysregulated function of the immune system affecting older people involves both innate and adaptive parts, with many mechanisms sharing molecular pathways implicated in the carcinogenesis process [37]. Among age-related alterations, the impairment of apoptotic cell death and the immunosuppressive role of some cytokines, such as interleukin-10 (IL-10) and transforming growth factor-β (TGF-β) which increasing in the elderly, could be relevant in the relationship between ageing and risk of tumor development [38, 39]. Therefore, immunosenescence may be linked to immune tolerance and contribute to an increased incidence of cancer with age.

Some evidence suggests that tumor-induced immunosuppression, through active mechanisms that can avoid or evade immune attack, might be more effective in ageing. Regarding the Fas ligand/Fas receptor (FasL/FasR) mechanism, the increased FasR expression observed in aged leukocytes might facilitate the immune escape of tumors expressing FasL [40]. A further example is the release of immunosuppressive cytokines by tumor cells such as TGF- β, IL-10 and others, that can suppress T cell responses and, in old subjects, may synergize with immunosuppressive cytokines overproduced by aged leukocytes [38].

## Immunosenescence and non-Hodgkin lymphoma

### Non-Hodgkin lymphoma and aging

The current available data focuses on B cell Non Hodgkin Lymphomas (NHL), which represent more than 90% of lymphoid neoplasms worldwide [41]. They are a heterogeneous group of clonal tumors of mature B cells that have distinctive clinical and biological behaviors [42–44]. NHL subtypes tend to mimic stages of normal B cell differentiation so that they can be classified according to the corresponding normal stage. In the context of NHL, a stepwise increase in the incidence of DLBCL over the last 20 years has been observed, particularly for patients older than 65 years [45]. The current increase in life expectancy naturally results in a higher number of elderly patients. Between lymphoma and aging, a complex interplay can be described [46]. B cell NHLs develop by a multistep process closely related to normal B cell counterpart that can be favored with aging [47]. Many potential factors can play a role in lymphomagenesis in the elders. As with all other cancer types, chronological ageing is associated with the accumulation of DNA damage particularly in stem cells [48]. Recently, significant large scale studies by whole-exome sequencing data have reported age-related clonal hematopoiesis, with somatic mutations in genes that are recurrently mutated in hematological neoplasms [49–51]. Also, epigenetic abnormalities that have a role in lymphoma development as in leukemia can accumulate with aging [52].

### Immunosenescence, chronic infection and lymphomagenesis in non-Hodgkin lymphoma of the elderly

In addition to abnormal genetic events, also age-related impairment in cancer protection is expected to promote B cell lymphomagenesis. The phenotype called “immunosenescence” is associated with a complex dysfunction that increases sensitivity to infections. Chronic infection with Cytomegalovirus (CMV) and EBV in the elderly caused by restricted T cell response can alter the B cell immune repertoire, leading to infection-linked diseases as well as some types of lymphoma [53]. It is known how B lymphomagenesis can be driven by microbial pathogens through chronic antigenic stimulation, and several examples are available in this regard. Some lymphotropic oncogenic viruses, EBV, Human-Herpesvirus-8 (HHV8), Human T-lymphotropic virus HTLV1) are directly responsible for lymphoid cell neoplastic transformation and are causative agents of different histologic entities, with varying aggressiveness. Other pathogens, indirectly via chronic inflammation of the mucosa-associated lymphoid tissue (MALT), have been associated with MALT Marginal Zone Lymphoma in various organs: Helicobacter pyloric, Campylobacter jejeuni, Chlamydia psittaci and Borrelia burgdorferi [54, 55]. Also, a causal relationship between Hepatitis C Virus (HCV) and NHL has been demonstrated and the most plausible molecular mechanism is lymphoma development by continuous antigenic stimulation. However, an increased incidence of NHL hystotypes linked to infections in the elderly has not been reported. A specific provisional entity in 2008’s World Health Organization (WHO) classification of tumors of hematopoietic and lymphoid tissue has been described as “EBV-positive DLBCL of the elderly” (EBV-DLBCL-E) [56, 57]. This lymphoproliferative disease occurs in absence of any primary or secondary immune deficiency and tends to have a post germinative center (GC)-phenotype and a poor prognosis [58]. The possible pathogenetic mechanism is lymphoma development as a consequence of immunosenescence and as part of the normal aging process, with a reduction in T cell repertoire. Immune modification can facilitate a second genetic event favored by EBV chronic infection in genetically instable B cell compartment. EBV-positive DLBCL thus represents a significant proof of the complex interplay between immunosenescence and lymphoma, and supports the leading role played by viruses in this setting. In 2016’s updated WHO classification of lymphoid neoplasia, this entity is now recognized as definite and the term “elderly” has been substituted by “not otherwise specified” (NOS) in such that these lymphomas can be present in younger patients as well [59]. In this patient group, EBV-positive DLBCLs usually have a positive outcome in contrast to EBV-DLBCL-E [60]. Nevertheless, EBV-DLBCL-E is a relatively rare lymphoid malignancy (< 5% in Western countries) and cannot explain the increased incidence of B-NHL in older patients.

### Diffuse large B cell lymphoma and aging-related molecular changes

Few studies have investigated whether a signature of aging can be seen in B cell NHL. Histopathologic features of DLBCL do not differ between age groups. However, studies of gene expression profiling (GEP) have described a higher frequency of activated B cell (ABC) DLBCL subtype in the elderly together with an increased B-cell lymphoma-2 (BCL2) expression and more genomic abnormalities [61–63]. These specificities can provide a basic understanding of the biological and clinical characteristics as well as the worst prognosis of DBCL in older patients.

Potential molecular alterations based on age in DLBCL patients have been described. Beheshti et al. identified significant age-related molecular changes after examining global transcriptome DLBCL data from The Cancer Genome Atlas, and striking transcriptional differences were associated with decreased metabolism and telomere dysfunction. The greatest functional changes occurring in older populations were related to key genes that strongly regulate the immune system [64]. These molecular factors influence tumor size and tumor progression in older DLBCL patients, but it is not clear if these findings may improve our understanding of lymphomagenesis as well as the role of immunosenescence.

## Immunosenescence and Hodgkin’s lymphoma

### Epidemiology and biology of Hodgkin lymphoma

Classical Hodgkin Lymphomas (cHL) are unusual B cell-derived malignancies and the majority of them manifest clinically in young adults, even though a bimodal age curve is described, with a second peak later in life. They account for 30% of all lymphomas. In contrast with NHL, their absolute incidence has not apparently changed and no increase has been observed in aged population [65, 66].

Neoplastic tissues include rare malignant Hodgkin and Reed-Stenberg (HRS) cells within an extensive but ineffective inflammatory/immune cell infiltrate composed of macrophages, eosinophils, mast cells and T cells [67]. Although HRS cells have lost expression of certain B cell surface proteins and, particularly, B cell receptors, they do not die by apoptosis, so alternative pathways for survival and growth are exploited [68]. Key strategies leverage different mechanisms, such as nuclear factor kappa-light-chain-enhancer of activated B cells (NF-κB) signaling, Janus kinase/signal transducers and activators of transcription (JAK/STAT) pathway, AP-1 transcription factor, tumor necrosis factor (TNF) receptor family protein expression but latest data support the major role of immune evasion [69–72]. Malignant HRS cells escape immune attack using multiple stratagems, including enhanced PD-1 signalling, secretion of soluble factors with inhibitory effects such as IL-10 and recruitment of abundant immunosuppressive C-C chemokine receptor type 4 regulatory T-cells (CCR4 Tregs) [73–75]. These features are highly informative for the position of immunosurveillance to tumor cells in cHL pathogenesis.

### Epstein-Barr virus and lymphomagenesis of Hodgkin lymphoma in the elderly

The biology and origin of cHL can be related to the oncogenic role of EBV [57]. EBV is a human virus, etiologically linked to a significantly wide range of lymphoproliferative diseases of B, T and NK cells. The role of EBV as growth-transforming agent is not linked to a single and simple oncogenic mechanism, but to a complex interplay between different patterns of viral gene expression, cellular genetic changes and immunity of the host. cHL is one of the major B cell malignancy types linked to EBV although it can occur in EBV-positive and negative form, both in apparently immunocompetent subjects [76]. But virus-specific immune surveillance should not be viewed as secondary. Indeed, immune impairment enhances lymphoma risk and the incidence of lymphoma and proportion of cHL cases that are EBV-positive are increased in Human Immunodeficiency Virus (HIV)-infected individuals. Furthermore, the proportion of cHL cases associated to EBV varies significantly with age and is more prevalent in older adults. The cases arising in the elderly appear as a different disease compared to cHL in young patients [77]. There are more patients who are found in advanced stages with B-symptoms and poor performance status, outlining a different pattern of histology subtypes with mixed cellularity that appear more evident if compared with younger individuals. EBV-associated disease was recognized as a poor prognostic factor and was also associated with an advanced-stage lymphoma [78]. The peak of cHL in adults may be attributed, at least partially, to senescence of EBV immunity and to an increased viral load. The prominent role of immunosenescence compared to other aging-related factors can explain why the increase in incidence of diffuse large B cell lymphoma over the last decades due to aging population has not been reflected in the incidence of cHL, which appears to stay constant [79]. cHL of the elderly, because of morphological features and association with EBV, is probably similar to cHL during HIV infection. In HIV-infected people, the incidence of cHL is 10-fold higher than in the general population, and the development of HIV-related cHL is not dependent on profound T-cell depletion, but only a modest impairment of CD4 lymphocytes is sufficient to elevate EBV viral load in the B cell system [80]. Indeed, after the introduction of Highly Active Antiretroviral Therapy (HAART) in the treatment of HIV infection, cHL incidence among HIV–infected cohorts has slightly increased [81, 82]. These common features between lymphomagenesis of cHL in older patients and in HIV-infected subjects reflect many similarities between biology of immunosenescence in aging and residual immunological defects in treated HIV infection [83]. Indeed, long-term therapy with HAART is associated with an increased risk of complications with degenerative nature and of cancer types that are similar to those observed among the elderly [84].

## Other links between immunosenescence and lymphoma in the elderly

In addition to the role of immunosenescence in the pathogenesis of lymphomas, still little is known about the interactions between aging immune system changes and other aspects of lymphoma biology as well as lymphoma management. Recently, some reports have emerged on the possible role of immunosenescence in the progression of cancer; furthermore, immunosenescence-associated changes are exacerbated by chemotherapy. Older patients’ immune background could play a critical role for higher risk of infections as a side effect of chemotherapy. Immunosenescence might, therefore, contribute to negative outcomes in patients with lymphoma and could be a target for therapy.

Finally, whether, or to what extent, immunosenescence plays a role in response or toxicity to anti-neoplastic therapy with immune checkpoint inhibitors is still a matter of debate, representing a relevant unmet need.

## Conclusion

The phenomenon of immunosenescence plays an essential, but poorly defined, role in the development of lymphoma. Older people are a heterogeneous portion of population that experiences various degrees of ageing and immune system remodeling. In addition, the mechanism of lymphomagenesis might vary by the type of lymphoma considered. In this setting, impairment of immune functions can predispose to development of lymphoma related to oncovirus infection, through reduced ability to clear infectious agents, chronic antigenic stimulation, lymphoma growth, immune evasion. EBV plays a central role in the onset of neoplastic lymphoproliferation, associated with immunological changes in aging. Current clinical and epidemiological findings, confirmed by molecular evidence, have generally revealed the oncogenic role of EBV only in a small size sample of lymphoma subtypes of elderly: EBV- DLBCL-E and cHL [85–87].

Since aging, as well as immunosenescence, is heterogeneous, both inter-individually and intra-individually, the assessment of this decline should be done individually and adapted not solely on chronological age. Currently, aging assessment is clinical and based upon a geriatric evaluation [88–90]. The challenging question is to find key biomarkers of immunaging that can help to better estimate the role of the aging phenotype and to include a measure of immunosenescence in studies by collecting data on elderly patients with cHL and EBV-DLBCL [91]. Thus far, of all the age-associated immune parameters found to be informative, it is not possible to select those that are crucial for clinical relevance.

Looking to the future, the new understanding of immunosenescence will have an impact on the treatment of lymphomas to further individualize therapy. Lastly, for research agenda, HIV-infected patients receiving HAART provide a model for a better understanding of how immune dysfunction with limited immune deficiency can promote lymphoma development [92].

## Abbreviations

(ABC) subtype: Activated B cell subtype
(GC)-phenotype: Germinative center phenotype
BCL2: B-cell lymphoma-2
CCR4 Tregs: C-C chemokine receptor type 4 regulatory T-cells
cHL: Classical Hodgkin Lymphomas
CMV: Cytomegalovirus
DLBCL: Diffuse Large B-cell Lymphoma
DLBCL, NOS: Diffuse Large B-cell Lymphoma, not otherwise specified
EBV: Epstein Barr Virus
EBV-DLBCL-E: EBV-positive DLBCL of the elderly
FasL: Fas ligand
FasR: Fas receptor
GEP: Gene expression profiling
HAART: Highly Active Antiretroviral Therapy
HCV: Hepatitis C Virus
HHV8: Human-Herpesvirus-8
HIV: Human Immunodeficiency Virus
HL: Hodgkin lymphoma
HRS cells: Hodgkin and Reed-Stenberg
HTLV1: Human T-lymphotropic virus
IL-10: Interleukin-10
JAK/STAT: Janus kinase/signal transducers and activators of transcription
MALT: Mucosa-associated lymphoid tissue
NF-κB: Nuclear factor kappa-light-chain-enhancer of activated B cells
NHL: Non Hodgkin lymphoma
NK cells: Natural killer cells
TCR: T-cell receptor
TGF-β: Transforming growth factor-β
TNF: Tumor necrosis factor
WHO: World Health Organization
